# First record of the aphid genus *Neonipponaphis* Takahashi (Hemiptera, Aphididae, Hormaphidinae) from China, with a description of one new species

**DOI:** 10.3897/zookeys.236.4068

**Published:** 2012-11-02

**Authors:** Jing Chen, Ge-Xia Qiao

**Affiliations:** 1 Key Laboratory of Zoological Systematics and Evolution, Institute of Zoology, Chinese Academy of Sciences, No. 1 Beichen West Road, Chaoyang District, Beijing 100101, P.R.China Institute of Zoology, Chinese Academy of Sciences Beijing China; 2 University of Chinese Academy of Sciences, No. 19 Yuquan Road, Shijingshan District, Beijing 100049, P.R.China

**Keywords:** *Neonipponaphis*, Aphididae, new record genus, new species, China

## Abstract

The aphid genus *Neonipponaphis* Takahashi is reviewed and reported in China for the first time, with a description of one new species, *Neonipponaphis pustulosis***sp. n.** on *Castanopsis eyrei* from Fujian. A key to species, morphological descriptions, features, host plants, and distributions are provided. Holotype and paratypes are deposited in the National Zoological Museum of China, Institute of Zoology, Chinese Academy of Sciences, Beijing, China.

## Introduction

The aphid genus *Neonipponaphis* was erected by Takahashi (1962), with description of type species *Neonipponaphis shiiae*. It was distinguished by prosoma of apterae distinctly separated from abdominal segments II–VII, bearing numerous fine setae and abdominal tergite VIII with 4–6 setae, as well as abdomen of alatae with many dorsal setae and siphunculi large in diameter at base, with distinct minute papillae. Until now, this genus is only known by the type species which occurs in Japan (Takahashi 1962; Ghosh and Raychaudhuri 1973; Blackman and Eastop 1994; Remaudière and Remaudière 1997). After identifying the specimens from Fujian, China and checking the specimens of the type species, we report a new species of *Neonipponaphis* from China, *Neonipponaphis pustulosis* sp. n., feeding on *Castanopsis eyrei*.

## Materials and methods

Specimens of the new species were collected from Mount Wuyi (Wuyishan City) by J. Chen, Q. H. Liu, and X. T. Li.

Aphid terminology in this paper generally follows Takahashi (1962). The unit of measurements in this paper is millimeters (mm).

In Table 1, the following abbreviations have been used: Ant.I, Ant.II, and Ant.IIIb, for antennal segments I, II, and the base of antennal segment III, respectively; PT, processus terminalis; Ant.IIIBW, basal width of antennal segment III; URS, ultimate rostral segment; BW URS, basal width of ultimate rostral segment; 2HT, second hind tarsal segment; MW Hind tibia, mid-width of hind tibia; BW Cauda, basal width of cauda; AP, anal plate; GP, genital plate.

**Table 1.** Morphometric data of species of *Neonipponaphis* (in mm).

Parts<br/> (For abbreviations see Materials and methods)		*Neonipponaphis pustulosis* sp. n.			*Neonipponaphis shiiae* Takahashi		
		Apterous vivipara (n=14)			Apterous vivipara (n=10)		
		Mean	Range	Standard Deviation	Mean	Range	Standard Deviation
Length (mm)	Body length	1.532	1.406–1.628	0.062	1.169	1.114–1.238	0.049
	Body width	1.388	1.184–1.628	0.126	1.058	0.960–1.171	0.074
	Whole antenna	0.242	0.221–0.259	0.011	0.211	0.187–0.221	0.010
	URS	0.078	0.072–0.086	0.005	0.071	0.067–0.077	0.004
	Hind trochanter and femur	0.122	0.115–0.134	0.009	0.092	0.077–0.096	0.007
	Hind tibia	0.160	0.144–0.173	0.013	0.112	0.106–0.115	0.006
	2HT	0.057	0.053–0.062	0.004	0.044	0.038–0.048	0.003
	Cauda	0.028	0.024–0.034	0.003	0.028	0.024–0.029	0.002
	BW Cauda	0.053	0.048–0.058	0.004	0.055	0.053–0.058	0.002
	Ant.IIIBW	0.022	0.019–0.026	0.002	0.019	0.017–0.022	0.001
	MW Hind tibia	0.034	0.029–0.036	0.003	0.027	0.026–0.029	0.001
	Cephalic setae	0.047	0.038–0.058	0.009	0.064	0.053–0.074	0.008
	Setae on Tergum I	0.068	0.060–0.079	0.008	0.073	0.058–0.091	0.010
	Setae on Tergum VIII	0.055	0.046–0.077	0.010	0.037	0.034–0.043	0.004
	Setae on Hind tibia	0.030	0.026–0.036	0.004	0.028	0.024–0.034	0.004
No. of setae on	Ant.I		1			1	
	Ant.II		2			2	
	Ant.IIIb		0			0	
	PT		0+3			0+3	
	URS		6			6	
	Terga II–VII		17–27			14–20	
	Tergum VIII		6–8			4–6	
	Cauda		7–10			8–10	
	Each lobe of AP		4–6			4–6	
	GP		14–18			14–19	
Ratio (times)	Whole antenna / Body	0.16	0.14–0.17	0.008	0.18	0.17–0.19	0.008
	Hind tibia / Body	0.11	0.10–0.11	0.007	0.10	0.09–0.10	0.009
	URS / BW URS	1.63	1.43–1.78	0.173	1.24	1.17–1.45	0.102
	URS / 2HT	1.43	1.23–1.64	0.203	1.57	1.47–1.67	0.066
	Cauda / BW Cauda	0.54	0.48–0.64	0.058	0.50	0.45–0.55	0.040
	Cephalic setae / Ant.IIIBW	2.21	1.60–2.88	0.547	3.31	2.67–3.88	0.460
	Setae on Tergum I / Ant.IIIBW	3.00	2.27–3.75	0.676	3.77	3.00–4.75	0.572
	Setae on Tergum VIII / Ant.IIIBW	2.49	1.91–3.20	0.416	1.89	1.56–2.25	0.253
	Setae on Hind tibia / MW Hind tibia	0.88	0.79–1.00	0.102	1.00	0.91–1.17	0.120

Specimen depositories: all specimens studied are deposited in the National Zoological Museum of China, Institute of Zoology, Chinese Academy of Sciences, Beijing, China (NZMCAS).

## Taxonomy

### Neonipponaphis

Takahashi

DD4A1487-E3AE-FB3C-7E14-591EDFF21C3E

http://species-id.net/wiki/Neonipponaphis

Neonipponaphis Takahashi, 1962: 9. Type species: *Neonipponaphis shiiae* Takahashi, 1962; by monotypy.
Neonipponaphis Takahashi: Ghosh and Raychaudhuri 1973: 164; Blackman and Eastop 1994: 775; Remaudière and Remaudière 1997: 187; Nieto Nafría et al. 2011: 281.

#### Generic diagnosis.

In apterae, body round, flat, and strongly sclerotized. Prosoma consisting of fused head, thorax, and abdominal segment I; abdominal segments II–VII fused and distinctly separated from prosoma; abdominal segment VIII free. Dorsum of prosoma with scattered oval or irregular-shaped pustules and numerous fine setae; abdominal tergites II–VII with scattered shorter setae; each tergite with a pair of submarginal setae, setae on tergites V and VI shorter than setae on the other tergites; tergites II and VII each with a pair of spinal setae; abdominal tergite VIII with 4–8 setae. Eyes with 3 facets in apterae and compound in alatae. Antennae in apterae indistinctly 3-segmented, with primary rhinaria placed wide apart on the terminal segment, in alatae 5-segmented with annular secondary rhinaria. Rostrum short and thick. Ultimate rostral segment blunt wedge-shaped, with 2 pairs of primary setae and a pair of secondary setae. Legs normal, tibial setae long and fine, hind tibiae with several short peg-like setae on distal part; tarsi 2-segmented, claws normal, first tarsal chaetotaxy in apterae: 2, 2, 2. Abdomen with many long dorsal setae and 4 pairs of spiracles in alatae. Siphunculi in apterae small, pore-like, in alatae low but much expanded basally, with distinct minute papillae around the pore. Cauda knobbed and constricted at base. Anal plate bilobed. Wings dusky and reticulated; fore wings with pterostigma dark and broadly rounded at hind margin, media once branched; hind wings with 2 obliques.

#### Distribution.

Japan and here newly recorded from China (Fujian).

#### Host plants.

*Castanopsis cuspidata* and *Castanopsis eyrei*.

#### Comments.

This genus is related to *Nipponaphis* Pergande, sharing several characters such as body of apterae aleyrodiform, flattened dorsoventrally, consisting of three parts - prosoma, fused abdominal segments II–VII, and separate abdominal segment VIII; dorsum of prosoma with scattered pustules; abdominal tergites II–VII with 6 pairs of submarginal setae and a pair of posteromesial setae on abdominal tergite VII; siphunculi pore-like; tarsi normal, 2-segmented, with normal claws; abdomen of alatae with 4 pairs of spiracles, and median vein of fore wings once branched. *Neonipponaphis* is distinguished by abdominal tergites II–VII distinctly separated from prosoma and the presence of numerous fine setae on the dorsum of prosoma and abdominal tergites II-VII in apterae.

#### Key to species of *Neonipponaphis*

(Apterous viviparous females)

**Table:** 

1	Body large, 1.41–1.63 mm long, with longer hind legs, the hind tibiae being 0.10–0.11 times as long as body. Pustules on prosoma small. Spinal and submarginal setae on dorsum of prosoma distinctly longer, thicker, and stiffer than the scattered dorsal setae. Abdominal tergite VIII with 6–8 dorsal setae	*Neonipponaphis pustulosis* sp. n.
–	Body relatively small, 1.11–1.24 mm long, with shorter hind legs, the hind tibiae being 0.09–0.10 times as long as body. Pustules on prosoma large. Spinal and submarginal setae on dorsum of prosoma longer than the scattered dorsal setae but similar to the latter in thickness and hardness. Abdominal tergite VIII with 4–6 dorsal setae	*Neonipponaphis shiiae* Takahashi

### Neonipponaphis pustulosis sp. n.

AF3CFAD7-6CE0-7184-60C5-AEA8D591AE00

urn:lsid:zoobank.org:act:ED5004C9-48A1-4503-B6C3-60B8746CFC6C

http://species-id.net/wiki/Neonipponaphis_pustulosis

**Figures 1–8.**
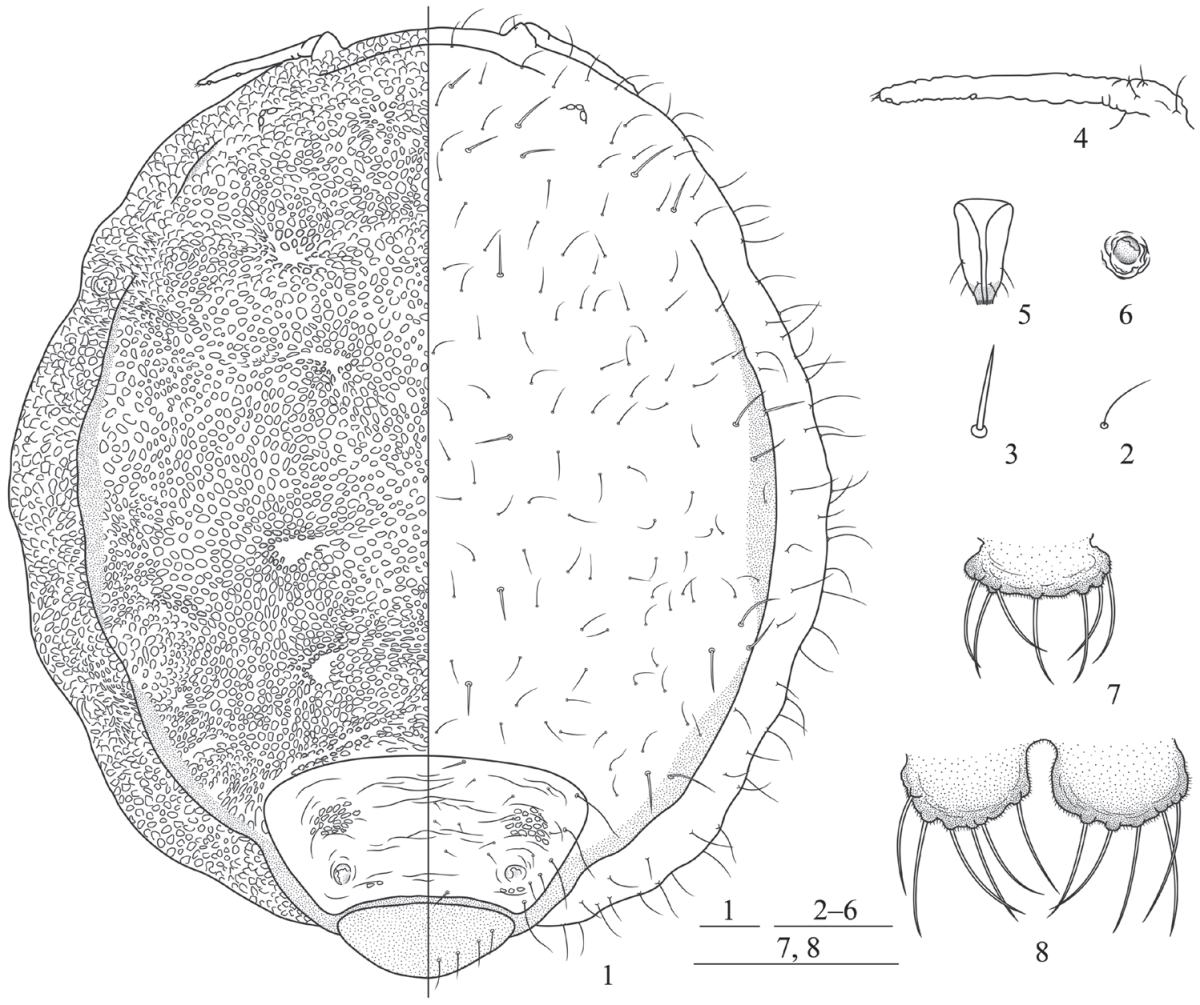
*Neonipponaphis pustulosis* sp. n. Apterous viviparous female: **1** dorsal view of body, with pustules in left and chaetotaxy in right **2** fine and pointed scattered dorsal seta **3** long, thick, and stiff dorsal seta **4** antenna **5** ultimate rostral segment **6** siphunculus **7** cauda **8** anal plate. Scale bars = 0.10 mm.

Figures 1 [Fig F2] –19

#### Locus typicus.

China (Fujian, 27.73279°N, 117.64512°E, altitude 1080 m).

#### Etymology.

The new species is named for the small and crowded pustules on the dorsum of prosoma. “*Pustulosis*” (Latin) means “blister, bubble”.

#### Description.

**Figures 9–17. F2:**
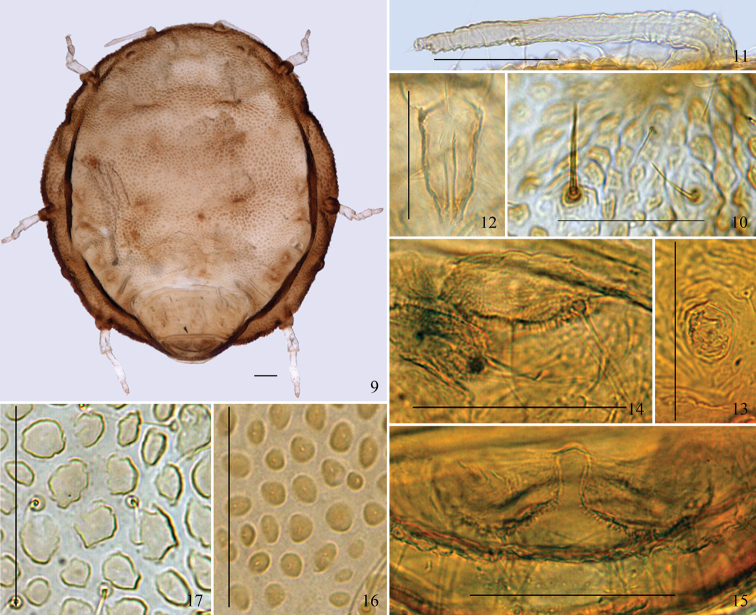
(**9–15**) *Neonipponaphis pustulosis* sp. n. Apterous viviparous female: **9** dorsal view of body **10** dorsal setae (long, thick, and stiff seta in left, fine and pointed seta in right) **11** antenna **12** ultimate rostral segment **13** siphunculus **14** cauda **15** anal plate. (**16–17**) Dorsal pustules on the same scale: **16** *Neonipponaphis pustulosis* sp. n. **17***Neonipponaphis shiiae* Takahashi. Scale bars = 0.10 mm.

**Figures 18–19.**
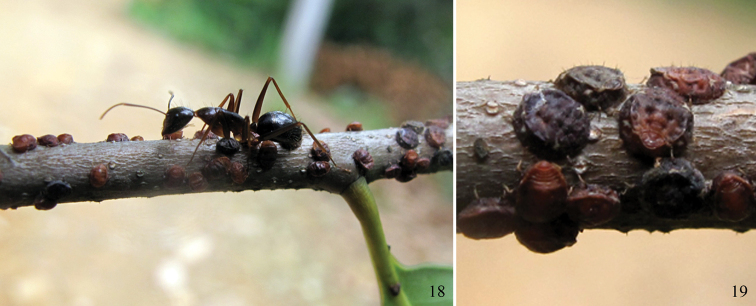
*Neonipponaphis pustulosis* sp. n. **18** a colony on the twig of *Castanopsis eyrei*, attended by an ant **19** apterous viviparous females in life.

*Apterous viviparous females*: Body round, flat, thickened, and strongly sclerotized (Figs 1, 9, 19). Reddish brown or blackish brown in life (Figs 18, 19). For morphometric data see Table 1.

#### Mounted specimens. 

Body brown; antennae and legs light brown. Prosoma consisting of fused head, thorax, and abdominal segment I; abdominal segments II–VII fused and distinctly separated from prosoma; abdominal segment VIII free (Figs 1, 9). Dorsum of prosoma with many oval or irregular-shaped pustules, small and crowded (Figs 1, 9, 16); pustules on vertical area of body similar, but those around the thoracic spiracles much smaller, protuberant, and conical in shape. Muscle attachment plates distinct, forming radial pattern with dorsal pustules (Figs 1, 9). Abdominal tergites II–VII wrinkled and with irregular oval markings (Fig. 1). Cauda (Figs 7, 14), anal plate (Figs 8, 15), and genital plate with spinulose sculptures. Dorsum of prosoma and marginal vertical area of body with numerous fine and pointed setae; head with a pair of cephalic setae, thick, stiff, and pointed; dorsum of prosoma with 13 pairs of submarginal setae, long, thick, and stiff, head dorsum with 3 pairs, pronotum with 2 pairs, mesonotum with 3 pairs, metanotum with 3 pairs, abdominal tergite I with 2 pairs; pro-, meso-, metanotum, and abdominal tergite I each with a pair of spinal setae, long, thick, and stiff; abdominal tergites II–VII with 17–27 scattered fine and pointed setae, shorter than dorsal setae on prosoma; tergites II–VII each with a pair of long submarginal setae, setae on tergites V and VI shorter; tergites II and VII each with a pair of spinal setae, stiff and pointed; tergite VIII with 6–8 dorsal setae (Fig. 1). Cephalic setae, marginal setae on abdominal tergite I, and dorsal setae on tergite VIII 1.60–2.88 times, 2.27–3.75 times, and 1.91–3.20 times as long as basal width of antennal segment III, respectively. Medial frons not protuberant (Figs 1, 9). Eyes 3-faceted (Fig. 1). Antennae short, indistinctly 3-segmented, 0.14–0.17 times as long as body (Figs 4, 11). Setae on antennae sparse; segments I–III each with 1, 2, 0+0 setae, respectively; processus terminalis with 3 apical setae. Primary rhinaria small, round, protuberant, and placed wide apart at the apex of terminal segment. Rostrum short and thick, not reading mid-coxae. Ultimate rostral segment blunt wedge-shaped, 1.43–1.78 times as long as its basal width, 1.23–1.64 times as long as second hind tarsal segment, with 2 pairs of primary setae and a pair of secondary setae (Figs 5, 12). Legs short, smooth, trochanter and femur fused (Fig. 9). Hind tibia 0.10–0.11 times as long as body. Setae on legs sparse, tibiae setae long and fine, hind tibiae with several short peg-like setae on distal part. Setae on hind tibia 0.79–1.00 times as long as its mid-width. First tarsal chaetotaxy: 2, 2, 2. Claws normal. Siphunculi small, pore-like, on abdominal tergite VI (Figs 6, 13). Cauda knobbed, constricted at base, 0.48–0.64 times as long as its basal width, with 7–10 setae (Figs 7, 14). Anal plate bilobed, each lobe with 4–6 setae (Figs 8, 15). Genital plate transversely oval, with two anterior setae and 12–16 setae along the posterior margin.

#### Specimens examined.

Holotype: apterous viviparous female, **CHINA:** Fujian (Wuyishan City, Xingcun Town, Mount Wuyi, 27.73279°N, 117.64512°E, altitude 1080 m), 11 Jun. 2011, No. 26868-1-3, on *Castanopsis eyrei*, coll. J. Chen, Q. H. Liu, and X. T. Li (NZMCAS). *Paratypes*: 13 apterous viviparous females, with the same collection data as holotype.

#### Taxonomic notes.

The new species is similar to the type species *Neonipponaphis shiiae* Takahashi, but differs in morphology by the characters given in the key.

#### Host plant.

*Castanopsis eyrei*.

#### Biology.

Apterous exules live on the twigs of the host plants and are attended by ants (Figs 18, 19). Other morphs and life cycle are unknown. Typical life cycle of nipponaphidines is host-alternating and holocyclic, with gall formation on *Distylium*. Thus, this species is either anholocyclic on *Castanopsis eyrei* or has gall-inhabiting generations still unknown or known under another name on *Distylium*. Field observations, transfer experiments, and molecular study are needed to elucidate its life cycle.

### Neonipponaphis shiiae

Takahashi

E03CA7D1-7185-82C6-D61C-A116614DA8F2

http://species-id.net/wiki/Neonipponaphis_shiiae

Figure 20

Neonipponaphis shiiae Takahashi, 1962: 9.
Neonipponaphis shiiae Takahashi: Blackman and Eastop 1994: 775; Remaudière and Remaudière 1997: 187.

#### Specimens examined.

10 apterous viviparous females, **JAPAN:** Gifu Prefecture, 20 Jul. 1968, No. E534, on *Castanopsis* sp., coll. M. Sorin (NZMCAS).

#### Distribution.

Japan.

#### Host plant.

*Castanopsis cuspidata*.

#### Biology. 

This species colonizes the branches and shoots of the host plants (Takahashi 1962).

**Figure 20.**
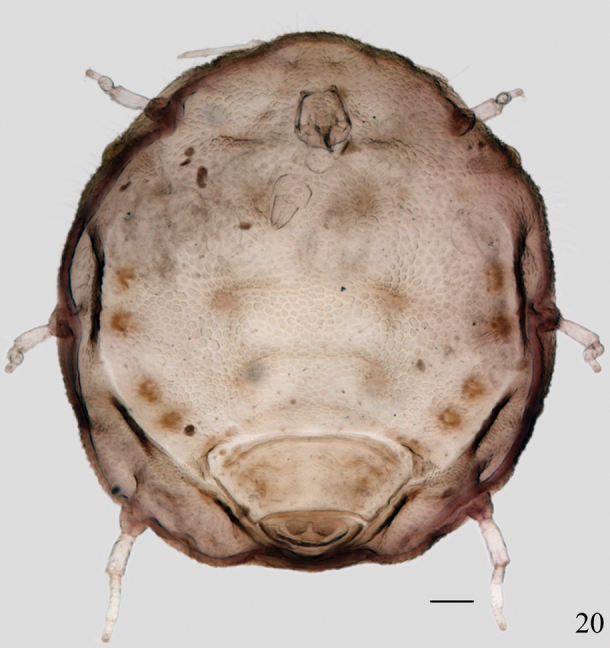
*Neonipponaphis shiiae* Takahashi. Apterous viviparous female, dorsal view of body. Scale bar = 0.10 mm.

## Supplementary Material

XML Treatment for Neonipponaphis

XML Treatment for Neonipponaphis pustulosis

XML Treatment for Neonipponaphis shiiae

## References

[B1] Blackman RL Eastop VF (1994) Aphids on the World’s Trees. An Identification and Information Guide. CAB International in Association with the Natural History Museum, Wallingford, 987 pp. http://www.aphidsonworldsplants.info [accessed 26.IX.2012]

[B2] Ghosh AK Raychaudhuri DN (1973) Studies on the aphids (Homoptera: Aphididae) from eastern India XV. A study of *Nipponaphis* Pergande and related genera with descriptions of a new genus and eight new species from eastern India Part I. Kontyû 41: 148-165.

[B3] Nieto Nafría JM Favret C Akimoto S Barbagallo S Chakrabarti S Mier Durante MP Miller GL Qiao G Sano M Pérez Hidalgo N Stekolshchikov AV Wegierek P (2011) Register of genus-group taxa of Aphidoidea. In: Nieto Nafría JM Favret C (Eds). Registers of Family-Group and Genus-Group Taxa of Aphidoidea (Hemiptera Sternorrhyncha).Universidad der León, León: 81-404.

[B4] Remaudière G Remaudière M (1997) Catalogue of the World’s Aphididae. Institut National de la Recherche Agronomique, Paris, 473 pp.

[B5] Takahashi R (1962) Aphids causing galls on *Distylium racemosum* in Japan, with descriptions of two new related species (Aphididae, Homoptera). Bulletin of the University of Osaka Prefecture, Series B 13: 1–11. http://www.bioenv.osakafu-u.ac.jp/bulletin/v13/v13_03.pdf [accessed 26.IX.2012]

